# The effects of gut microbiota-derived short-chain fatty acids (SCFAs) on human colon cancer cells: An in vitro study

**DOI:** 10.1016/j.jgeb.2025.100610

**Published:** 2025-10-29

**Authors:** Shiva Darabi, Fatemeh Keshavarzi, Parviz Ashtari, Farahnaz Motamedi Sedeh, Behrouz Alirezapour

**Affiliations:** a Department of Biology, Sa.C., Islamic Azad University, Sanandaj, Iran; b Radiation Application Research School, Nuclear Science and Technology Research Institute (NSTRI), Moazzen Blvd., Rajaeeshahr, P.O. Box 31485-498, Karaj, Iran; c Department of Veterinary and Animal Diseases, Nuclear Agriculture Research School, Nuclear Science and Technology Research Institute, Karaj, Iran; d Radiation Application Research School, Nuclear Science and Technology Research Institute (NSTRI), Tehran, Iran

**Keywords:** Short-chain fatty acids, *Cyp1a1*, Colorectal cancer, Epigenetic change, Caco-2 cell line

## Abstract

Colorectal cancer (CRC) has always been a major concern for researchers due to its deadly nature. One of the microbial metabolites found in the gut is short-chain fatty acids (SCFAs), which have the ability to regulate the immune system and exert protective effects against CRC. In this study, we investigated the effects of SCFAs on the expression of the *CYP1A1* gene in Caco-2 cells at varying concentrations. The Caco-2 cell line and HDF control cell line were treated with three concentrations (7.5, 15, and 30 mM) of NaA(sodium acetate), NaB(sodium butyrate), and NaP(sodium propionate) for three different time periods (24, 48, and 72 h). Additionally, two concentrations (2.5 and 10 μM) of CH223191 (an AhR gene antagonist) alone and a concentration of 10 μM of CH223191 in combination with the mentioned concentrations of NaA, NaB and NaP were applied to the cells for 48 h. The gene expression of *CYP1A1* was examined using Real-time PCR. The expression of the *CYP1A1* gene increased with concentrations of NaA and NaB at all time points, and with 30 mM of NaP at 24 h (p < 0.05). However, increasing the concentration of NaB and NaP led to decreased expression of the *CYP1A1* gene. These findings suggest the potential use of SCFAs as epigenetic drugs for the prevention and treatment of CRC.

## 1 Introduction

Overall, there has been an observed increase in the prevalence of colorectal cancer(CRC) in Iran, with the incidence rising by 1.6 times compared to the global trend.^1^, ^2^, ^3^, ^4^, ^5^ Various biological defense mechanisms can modulate harmful interactions between environmental pollutants and cell functions, including DNA damage and repair, cell cycle adjustment, and levels of xenobiotic-metabolizing enzymes.^6^

The human gut microbiota, which consists of trillions of microorganisms, plays a vital role in maintaining host health.^7^ A balanced gut microbiome, often referred to as eubiosis, is essential for various physiological functions, including digestion, immune response, and metabolic processes.^8^ One of the key products of microbial fermentation in the gut is short-chain fatty acids (SCFAs), such as acetate, propionate, and butyrate. SCFAs have been shown to have numerous beneficial effects on host health, including anti-inflammatory properties, modulation of immune responses, and serving as energy sources for colonic epithelial cells.^9^

Conversely, dysbiosis, an imbalance in the gut microbiota composition can lead to a decrease in SCFA production and is associated with various health conditions, including obesity, diabetes, inflammatory bowel disease, and other metabolic disorders. Understanding the relationship between SCFAs and gut microbiota composition is essential for developing interventions aimed at restoring eubiosis and enhancing overall health.^10^, ^11^

Acetate plays an important role in enabling bifidobacteria to inhibit intestinal pathogens.^12^, ^13^ Additionally, butyrate serves as the primary source of energy for colon cells, with its oxidation accounting for approximately 60–70 % of oxygen consumption in the human colon.^14^

Butyrate stimulates intestinal epithelial cells, increasing mucin production, which leads to changes in bacterial adhesion^15^ and enhances the integrity of tight junctions. Once produced, SCFAs are absorbed and used in various biosynthetic pathways by the host.^16^, ^17^ Propionate, aids in gluconeogenesis, while acetate and butyrate, are involved in lipid biosynthesis. In other words, these three compounds play a role in metabolic control processes.^18^

While all three major intestinal SCFAs have protective effects on diet-induced obesity, butyrate and propionate have been found to have more pronounced effects than acetate.^19^, ^20^ Additionally, unlike acetate, butyrate and propionate, have been reported to inhibit gut hormone production and reduce food absorption.^13^ Studies focused on butyrate have demonstrated that SCFAs, especially butyrate, can provide resistance to the progression of colon cancer.^18^

All of these benefits are effective in inhibiting colon cancer. Sodium butyrate, a natural HDACi(inhibitor), increases the expression of cytochrome P450 1A1 (CYP1A1) in the metabolism of carcinogens.^21^ However, evidence of the effects of HDACi on CYP1A1 modulation is inconsistent, suggesting that the effects may be species- and tissue-dependent.^22^, ^23^ Microbiota can simultaneously produce both AhR(aryl hydrocarbon receptor) ligands and SCFAs. Acetate, butyrate and propionate increase AhR ligand-induced responses by inhibiting histone deacetylase^16^ and increasing the expression of the CYP1A1 gene.^24^, ^25^, ^26^, ^27^, ^28^, ^29^, ^30^

Since enterocytes are directly exposed to millimolar concentrations of SCFA, these metabolites can impact CYP1A1 levels and function. Research has shown that HDAC inhibition is species and tissue-specific.^29^, ^30^ Butyrate has significant effects on cell proliferation, cell differentiation, gene expression, and inhibition of HDACs to increase histone acetylation.^31^, ^32^ Treatment of Caco-2 cells with butyrate activates caspase3 and increases apoptosis.^33^, ^34^, ^35^ Additionally, treating human colonic epithelial cells with butyrate increases the expression of p21wf1/cip, which plays a role in cell cycle regulation, while simultaneously decreasing the expression of cyclin A1, thus influencing the cell cycle.^36^ Similar findings regarding butyrate were reported in studies conducted by Zapletal^37^ and Metidji.^38^

In this study, the effect of SCFAs on the expression of cytochrome P450 1A1 (CYP1A1) in Caco-2 cells was investigated at various concentrations.

## 2 Materials & Methods

### 2.1 Materials

All reagents and chemical solutions were purchased from Merck Company. All SCFAs including sodium acetate (NaA), sodium butyrate (NaB), sodium propionate (NaP) and CH223191 were purchased from Sigma Aldrich Company. The cells were derived from the Pasteur Institute in Iran. Materials for cell culture, were obtained from Gibco Company in Brazil. The cDNA Synthesis Kit (Cat. No: EX6101) and Protein extraction Kit (Cat. No:SK1151-50) were obtained from Sina Clone in Iran. The cells were cultured in DMEM/F-12 (Dulbecco's Modified Eagle Medium/Nutrient Mixture F-12)(Hy Clone ™, Thermo Scientific ™) including 20 % FBS(fetal bovine serum) (Hy Clone ™, Thermo Scientific ™), penicillin (50 U/ml), and streptomycin (50 U/ml).^39^ The cells were grown at 37 °C in an atmosphere(humidity 95 %, CO2 5 %). Cells were subcultured at a concentration of 10^6^ cells/ml the night before treatments. Cell counting was done using 0.4 % Trypan Blue and a Neubauer hemocytometer.^40^ Flowchart 1 displays the steps of the procedure used in the current study.

**Figure:**
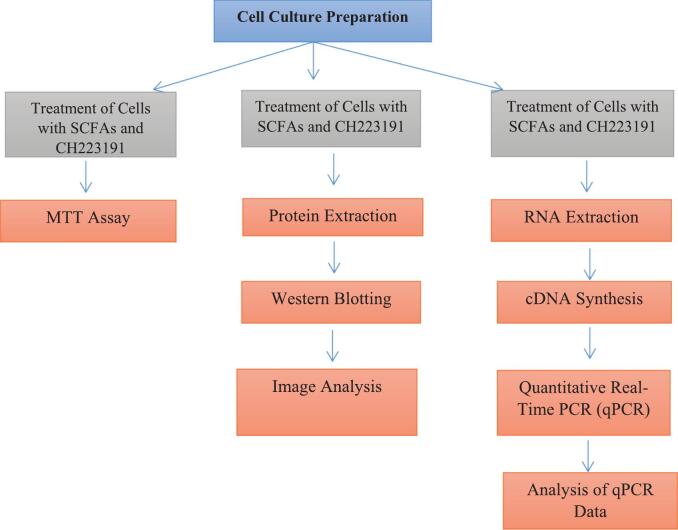

Flow chart 1. The steps of the procedure.

### 2.2 Cell treatment

Cells were treated at three different times with specified reagents: 7.5, 15, and 30 millimolar (mM) solutions of SCFAs, respectively. Cell viability was determined by counting live cells stained with the red exclusion dye, erythrosine B. Two concentrations of CH223191 (2.5 and 10 ÂµM (µM), an inhibitor of the AhR, were tested individually. The effects of CH223191 (10 µM) in combination with various concentrations of NaA, NaB and NaP were assessed after 48 h of incubation. Untreated cells, consisting only cell suspension and medium, were used as controls.

### 2.3 Treatment and MTT assay for viability analysis of cells

The MTT(3-(4,5-dimethylthiazol-2-yl)-2,5-diphenyltetrazolium bromide) tetrazolium reduction assay was used to investigate the potentiating effects of SCFAs on cytotoxicity in Caco-2 cells following the standard method.^41^ The cells were seeded in 96-well plates and allowed to adhere for 24 h. Subsequently, 1 × 10^6^ cells/mL of Caco-2 cells were seeded in each well of a 96-well plate and incubated for 24 h. The cells were then treated with different concentrations (7.5, 15 and 30 mM) of NaA, NaB, NaP and CH223191 (10 μM), and incubated for 24, 48 and 72 hrs. Wells without SCFAs were used as controls. After the incubation period, the culture medium was removed from the wells, and 200 µL of MTT reagent (5 mg/mL; ThermoFisher Scientific,) was added to each well and incubated for an additional four h. The contents of the wells were then aspirated, 200 µL of DMSO was added, and the plates were shaken for 20 min to dissolve the formazan crystals in isopropanol. The amount of formazan produced was measured by absorbance at 570 nm.

### 2.4 RNA Isolation

The cells were incubated in the presence of 10 mL of SCFAs, for 48 h. A total of 5 × 10^6^ cells were used for extraction. RNA extraction was then carried out from both treated and control cells using the Cytoplasmic & Nuclear RNA Purification Kit (Cinna Gen Inc., Iran) following the manufacturer’s instructions.

### 2.5 Synthesis of cDNA

The cDNA synthesis was conducted using the SinaClon-Iran cDNA synthesis kit following the manufacturer’s instructions. Initially, a combination of RNA and primers (10 μL) was prepared with extracted RNA (5 μg), random hexamers (1 μL), and DEPC water. This mixture was incubated for 5 min at 65 °C using a thermocycler, followed by chilling on ice for 2 min. To reach, a final volume of 20 µL, the cDNA synthesis mix was added, which included 5XBufferM-MuLV (4 µL), DTT (1 µL), M−MuLV reverse transcriptase (0.5 µL), RNase inhibitor (0.5 µL), 10 mM dNTP Mix (2 µL) and DEPC water. The mixture was then placed in a thermocycler and subjected to the following temperature conditions: 10 min at 25 °C, 60 min at 42 °C, and 5 min at 85 °C. A polymerase chain reaction (PCR) was carried out to assess the accuracy of the synthesized cDNA using GAPDH as a housekeeping gene. Forward (CT GAC TTC AAC AGC GAC ACC) and reverse (TAG CAA ATT CGT TGT CAT ACC) primer sequences for the GAPDH gene were prepared. The master mix 2X (12.5 μL) (SinaClon − Iran), 200 nM of each primer, and DEPC water were combined to make a final volume of 25 μL. The PCR conditions were as follows: initial denaturation at 94 °C for 2 min, 25 cycles of denaturation (94 °C, 30 S), annealing (52 °C, 45 S) and elongation (68 °C, 1 min), followed by a melting curve (68 °C, 7 min) and finally placed in the PCR device at 4 °C. The PCR products were then analyzed by electrophoresis on a 1 % agarose gel for 60 min at 100 V and were evaluated using GEL DOC XR + and Image Lab software.^40^, ^41^

### 2.6 Real-time PCR

Quantitative evaluation of the *CYP1A1* gene expression was performed twice for each sample. The reaction mixture included Sina SYBR Green HS-qPCR Mix (2x; Sina Clon − Iran), 12.5 μL (1x) of forward primer (5́-CGT GCT GCA GAT CCG AAT TG −3́) at 0.5 µL (0.1–600 nM), reverse primer (5́-GCT CAC ATG CTC TTC CAG GT −3́) at 0.5 µL(0.1–600 nM) (Metabion International AG), DNA template(1 pg-1 µg) and sterile water (up to 25 μL). The amplification program consisted of a preliminary denaturation stage at 95 °C for 2 min, followed by denaturation at 95 °C for 30 s, annealing at 56 °C for 30 s, and elongation at 72 °C for 30 s. This cycle was repeated 40 times. The melting curve was generated from 57-95 °C using MyGo Pro real-time PCR. The GAPDH gene was used as an internal control. Real-time PCR data were analyzed using the 2-ΔΔCT method and SPSS version 21 software.^40^, ^41^

### 2.7 Protein extraction

Protein extraction was carried out following the protocol (Arsam Farazist-Iran). The collected cells were washed twice with PBS. Subsequently, 1 mL of RIPA lysis buffer with an inhibitor was added and incubated for 30 min at 4 °C with vortexing. After that, the solution containing proteins was centrifuged, and the resulting supernatant was stored at −80 °C. The protein concentration was then measured using the Bradford method.^42^

### 2.8 Western blotting

Electrophoresis was performed using a 12 % polyacrylamide gel at 120 V for 100 min after protein extraction. The proteins were then transferred to a PVDF membrane in a Western Blot tank at 100 V and 4 °C for 90 min. The PVDF membrane was washed with water, followed by three washes with TBST. It was then gently shaken in a blocking buffer at room temperature for one h. Subsequently, the PVDF membrane was further shaken in 50 ml of blocking buffer and mixed with 10 μL of primary anti-CYP450 1A1 antibody (Sigma-Aldrich) at 4 °C for 16 h. After three more washes with TBST, the PVDF membrane was incubated with 4 μL of secondary goat anti-mouse IgG H&L (HRP) (Abcam-USA) dissolved in 20 ml blocking buffer (at a 1:5000 ratio) at room temperature for one h. The PVDF membrane was then re-washed three times with TBST and immunohistochemical staining was performed using DAB. For staining, the PVDF membrane was placed in 50 ml of staining solution and 50 μL of 30 % H2O2. The reaction was stopped with distilled water when the bands appeared. Finally, the PVDF membrane was photographed using a BIO-RAD Gel Doc.^42^

### 2.9 Statistics

An ANOVA test was used to analyze the gene expression changes across the different groups. Gene expression changes were calculated with Genex6 and statistical analysis was performed using SPSS version 21. The graphs were generated using GraphPad Prism version 8.

## 3 Results

The summary of the results from the various steps of the current study is presented in Flowchart 2.

**Figure:**
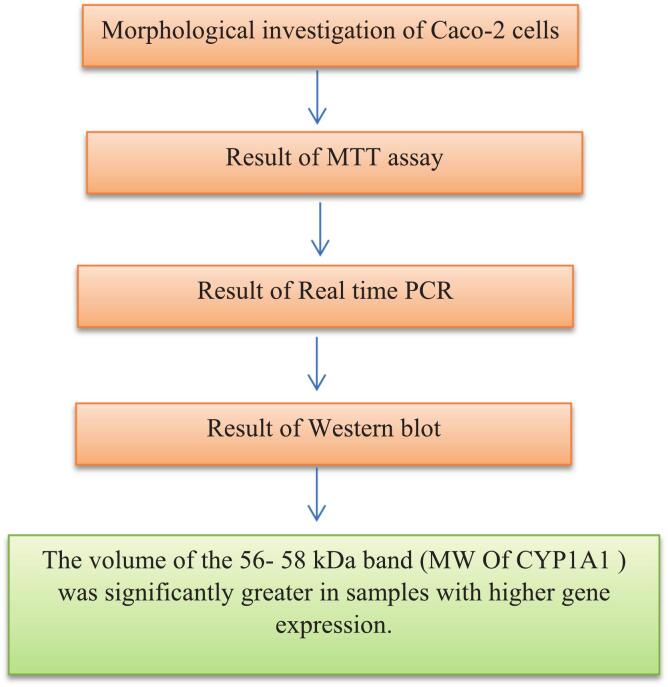

Flowchart 2. The summary of the results from the various steps of the current study

### 3.1 Morphological evaluation of Caco-2 cells after treatment compared to control cells

**Fig. 1:**
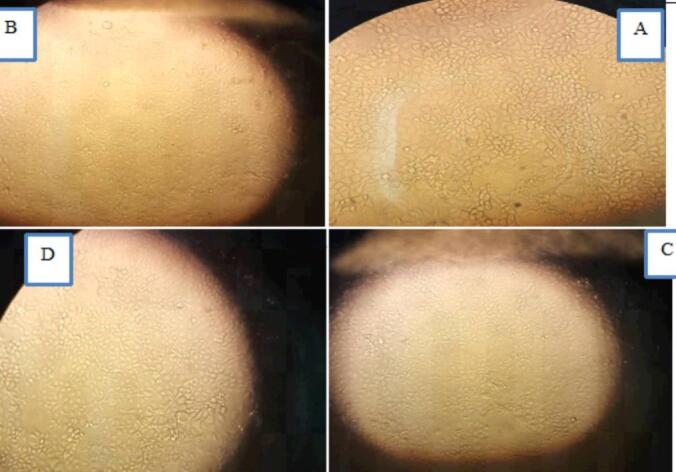
Caco-2 cells were treated with three different concentrations of NaA over a 72-h interval at the following concentrations: A. Untreated control cells, B. 30 mM, C. 15 mM, and D. 7.5 mM.

**Fig. 2:**
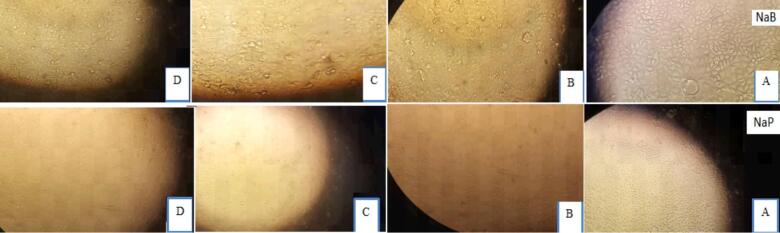
Caco-2 cells were treated with three different concentrations of NaB(above) and NaP(below) over a 72-h interval at the following concentrations: A. Untreated control cells, B. 30 mM, C. 15 mM, and D. 7.5 mM.

Isolated Caco-2 cells (human colon cancer cells) were exposed to NaA, NaB and NaP at three concentrations (7.5, 15 and 30 mM) for three-time intervals (24, 48 and 72 h). CH223191, an inhibitor of the AhR, was administered to two groups of cells at concentrations of 2.5 and 10 μM over a 48-h period. In the other nine groups of cells, a concentration of 10 μM of CH223191 was administered in combination with three concentrations (7.5, 15 and 30 mM) of NaA, NaB and NaP over a 48-h period. Control groups did not receive CH223191 or the SCFAs. The changes in the *CYP1A1* gene expression, morphology, proliferation and death rates of treated and untreated cells were then compared. Fig. 1, Fig. 2 show the morphology of Caco-2 cells treated with NaA at three different concentrations and 24 h and 72 h intervals. In the experiments, the impact of NaA on cell morphology and cell death was visually observed (Fig. 1).

In figure 2, the morphology of Caco-2 cells treated with NaB and NaP was observed at three different concentrations over 72 h intervals.

**Fig. 3:**
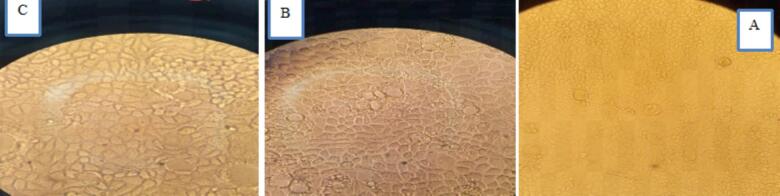
Caco-2 cells were treated at concentrations of 2.5 and 10 μM of CH223191 for 48 h. A] Untreated control. B] 2.5 μM concentration of CH223191 for 48 h. C] 10 μM concentration of CH223191 for 48 h.

Additionally, Fig. 3 depicts Caco-2 cells treated with concentrations of 2.5 and 10 μM of CH223191 for 48 h in comparison to control cells. The figure illustrates the morphological effects of the CH223191 antagonist on the AhR gene showcasing changes in cell morphology and cell death in Caco-2 cells at concentrations of 10 and 2.5 μM of CH223191.

### 3.2 Results MTT assay

**Fig. 4:**
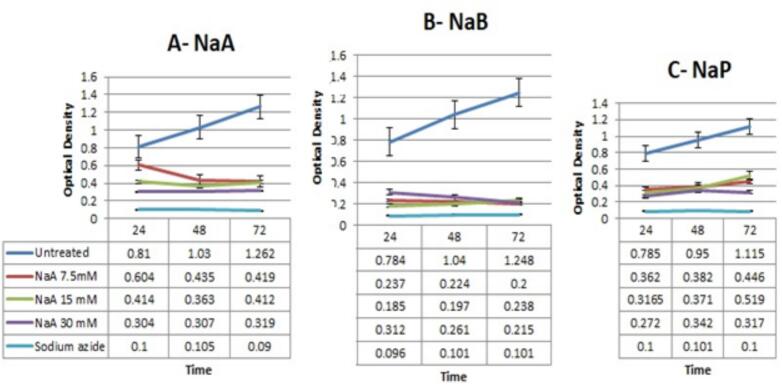
The results of the MTT assay analysis NaA, NaB and NaP on caco-2 cell lines.

The results of the MTT assay and ANOVA analysis on cell lines treated with SCFAs (NaA, NaB and NaP) compared to control cells are presented in Fig. 4 and Table 1. It is evident that the optical density (OD) absorption at a concentration of 30 mM NaA over three time periods was significantly reduced with a p-value of < 0.001 (Fig. 4-A). Fig. 4-B shows a significant decrease in OD rate at a concentration of 15 mM NaB in the 24 and 48 h time periods, as well as at a concentration of 7.5 mM NaB in the 72-h time period, with a p-value of < 0.001. Fig. 4-C demonstrates that the OD at a concentration of 30 mM NaP over a 24-h period was significantly lower with a p- value of < 0.001 (Table 1).

**Table 1:** The results of the ANOVA analysis on treated cell lines.

*Source of Variation*	*SS*	*df*	*MS*	*F*	*P-value*	*F crit*
Between Groups [NaA]	4.7752948	14	0.341092	41261.1878	2.92293E-60	2.03742044
Within Groups	0.000248	30	8.27E-06			
Total	4.7755428	44				
Between Groups [NaB]	5.407944066	14	0.386281719	1271.86848	1.3113E-37	2.03742044
Within Groups	0.00911136	30	0.000303712			
Total	5.417055426	44				
Between Groups [NaP]	3.792908311	14	0.270922	3851.9719	8.113E-45	2.03742044
Within Groups	0.00211	30	7.03E-05			
Total	3.795018311	44				

**Fig. 5:**
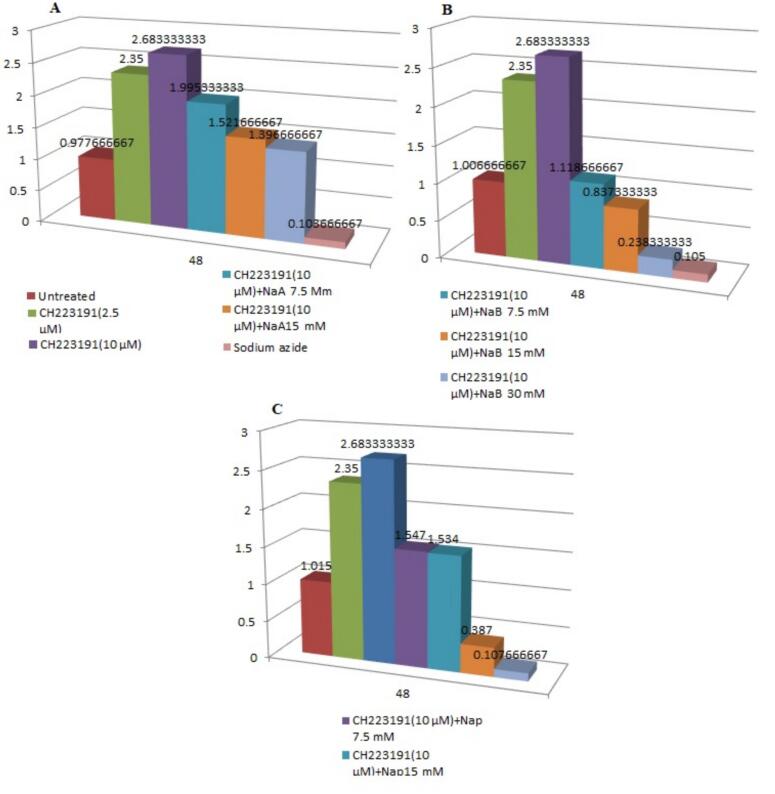
The results of the MTT assay analysis on treated cell lines compared with an inhibitor [CH223191] and SCFAs compared to control cells.

Also, the results of the MTT assay and ANOVA analysis on treated cell lines with an inhibitor (CH223191) and SCFAs compared to control cells are presented in figure 5 and Table 2.

**Table 2:** The results of the ANOVA analysis on treated cell lines compared with an inhibitor [CH223191] and SCFAs compared to control cells.

*Source of Variation*	*SS*	*df*	*MS*	*F*	*P-value*	*F crit*
Between Groups [NaA + CH223191]	13.68594724	6	2.2809912	524.8829	1.1881E-15	2.847725996
Within Groups	0.06084	14	0.0043457			
Total	13.74678724	20				
Between Groups [NaB + CH223191]	17.46479933	6	2.91079989	717.787666	1.34226E-16	2.847725996
Within Groups	0.056773333	14	0.00405524			
Total	17.52157267	20				
Between Groups [NaP + CH223191]	16.28731124	6	2.7145519	642.743788	2.89805E-16	2.847725996
Within Groups	0.059127333	14	0.0042234			
Total	16.34643857	20				

In figure 5-A, it is shown that the OD in the CH223191 sample at a concentration of 10 µM, as well as the CH223191 sample at a concentration of 10 µM in combination with a concentration of 7.5 mM NaA increased significantly over a 48-h period (p < 0.001). Fig. 5-B shows a significant increase in OD in the CH223191 sample at a concentration of 10 µM, as well as in the CH223191 sample at a concentration of 10 µM in combination with a concentration of 30 mM NaB in the 48-h time frame with p < 0.001. Fig. 5-C illustrates that the OD in the CH223191 sample at a concentration of 10 µM, as well as the CH223191 sample at a concentration of 10 µM in combination with concentrations of 7.5 and 30 mM NaP over a 48-h period was significantly higher with a p < 0.001.

### 3.3 Results of real-time PCR

#### 3.3.1 The gene expression curves of *CYP1A1* were measured in Caco-2 cells

**Fig. 6:**
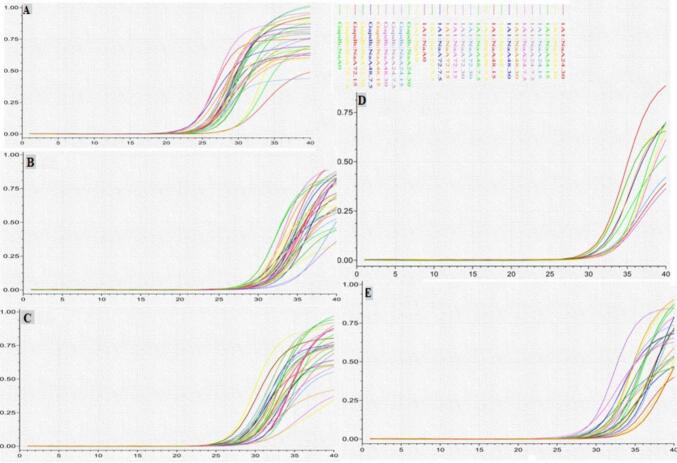
The gene expression curves of *CYP1A1* were measured in Caco-2 cells treated with three concentrations (7.5, 15, and 30 mM) of NaA (A), NaB (B), and NaP (C) for three time periods (24, 48, and 72 h). Additionally, cells were treated with a concentration of 10 μM of CH223191 alone(D), a combination of 10 μM of CH223191 with three concentrations of NaA for 48 h and a combination of 10 μM of CH223191 with three concentrations of NaB and NaP for48 h (E). The GAPDH gene was used as a positive internal control, cDNA of untreated samples as a positive external control, and samples without cDNA as a negative external control.

Fig. 6 shows the amplification curves of the *CYP1A1* gene in the RNAs extracted from treatment cells with all three fatty acids NaA, NaB and NaP at concentrations of 7.5, 15, and 30 mM. The housekeeping gene GAPDH was used as a positive internal control, the cDNA of the untreated sample was used as a positive external control, and the sample without cDNA was used as a negative external control. Treating Caco-2 cells with NaA, NaB, and NaP resulted in changes in the expression of the *CYP1A1* gene after 48 h.

#### 3.3.2 Expression changes of *CYP1A1* genes, separately, in cells treated with NaA, NaB, and NaP

**Fig. 7:**
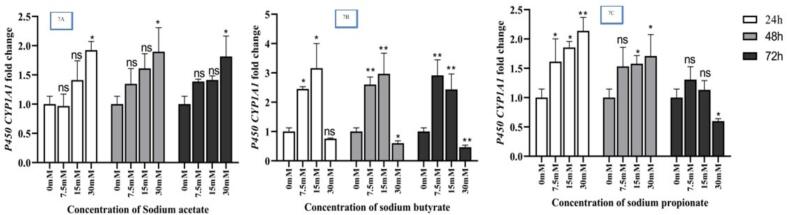
Expression changes of *CYP1A1* genes in cells treated separately with NaA (7A), NaB (7B), and NaP (7C) at concentration of 7.5, 15, and 30 mM for 24, 48 and 72 h, respectively. Results (mean ± SD) were calculated as fold of control values. Significant levels were denoted as *p < 0.05, **p < 0.01 and ***p < 0.001, based on ANOVA.

The gene expression of *CYP1A1* was altered by different concentrations (7.5, 15, and 30 mM) of NaA(7A), NaB(7B) and NaP(7C) at different time intervals (24,48 and 72 h) (Fig. 7).

#### 3.3.3 Comparing expression of *CYP1A1* gene in the cells treated with NaA, NaB, and NaP at different times

**Fig. 8:**
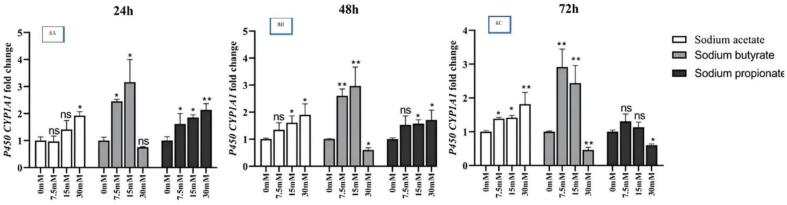
Comparing expression of the *CYP1A1* gene in the treated cells by NaA, NaB, and NaP at different times,8A. 3 mentioned SCFAs in 3 concentrations (7.5, 15 and 30 mM) over a period of 24 h; 8B. the same 3 SCFAs in the same 3 concentrations (5, 15 and 30 mM) over a period of 48 h; 8C. the same SCFAs in 3 concentrations (7.5, 15 and 30 mM) over a period of 72 h, as well as control samples. Results (mean ± SD) were calculated as fold of control values. *p < 0.05, **p < 0.01 and ***p < 0.001, ANOVA.

Fig. 8A shows that over a 24 h period, concentrations of 7.5 mM and 15 mM of NaB (*P <* 0.05) resulted in a significant increase in *CYP1A1* gene expression. Also, concentrations of 7.5 mM, 15 mM and 30 mM of NaP as well as 30 mM of NaA also increased the expression of the *CYP1A1* gene (*P <* 0.05). However, during this period, a concentration of 30 mM NaB reduced the expression of the *CYP1A1* gene. Fig. 8B shows that over a 48 h period, concentrations of 7.5 mM and 15 mM NaB (*P <* 0.05) resulted in a significant increase in *CYP1A1* gene expression. Concentrations of 15 mM and 30 mM NaA, as well as 7.5 mM, 15 mM and 30 mM NaP (*P <* 0.05) also increased the expression of the *CYP1A1* gene. However, during this period, a concentration of 30 mM NaB decreased the expression of the *CYP1A1* gene. Fig. 8C shows that over a 72 h period, concentrations of 7.5 mM and 15 mM NaB (*P <* 0.05) resulted in a significant increase in the *CYP1A1* gene expression. Also, concentrations of 7.5 mM, 15 mM and 30 mM NaA (*P <* 0.05) also increased the expression of the *CYP1A1* gene. However, during this period, concentrations of 30 mM NaB and NaP decreased the expression of the *CYP1A1* gene. The analysis results comparing the expression of the *CYP1A1* gene in treated cells with NaA, NaB, and NaP at different times are presented in Table3. The analysis was conducted using ANOVA.

**Table 3:** Analysis results comparing expression of the *CYP1A1* gene in the treated cells with NaA, NaB, and NaP at different times, by ANOVA analysis.

NaA	Df	Sum.Sq	Mean.Sq	F.value	p
Time	2.00000	0.10866	0.05433	1.13510	0.35359
Concentration	3.00000	2.71840	0.90613	18.93160	0.00008
interaction	6.00000	0.27804	0.04634	0.96820	0.48590
Residuals	12.00000	0.57436	0.04786		
NaB					
Time	2.00000	0.17660	0.08830	0.95900	0.41093
Concentration	3.00000	21.76970	7.25660	78.76700	0.001
interaction	6.00000	0.54610	0.09100	0.98800	0.47481
Residuals	12.00000	1.10550	0.09210		
Nap					
Time	2.00000	2.24886	1.12443	25.92050	0.00004
Concentration	3.00000	1.30172	0.43391	10.00250	0.00138
interaction	6.00000	2.23394	0.37232	8.58290	0.00090
Residuals	12.00000	0.52056	0.04338		

### 3.4 Comparing expression of the *CYP1A1* gene in the cells treated with NaA, NaB, and NaP along inhibitor of CH223191

**Fig. 9:**
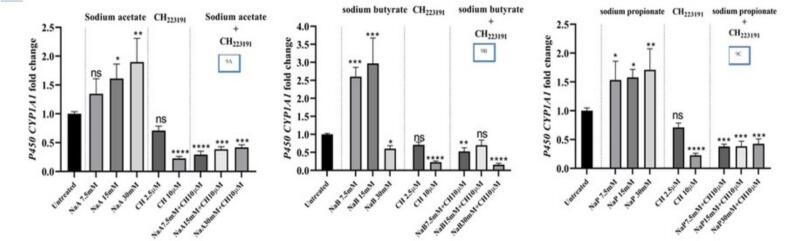
Comparing expression of the *CYP1A1* gene in the cells treated with NaA, NaB, and NaP along inhibitor of CH223191; 9A] concentrations of 7.5, 15 and 30 mM of NaA, as well as 2.5 and 10 μM of CH223191 and a combination of the above concentrations of NaA with 10 μM of CH223191; 9B] concentrations of 7.5, 15 and 30 mM of NaB, as well as 2.5 and 10 μM of CH223191 and a combination of the above concentrations of NaB with 10 μM of CH223191; 9C] concentrations [7.5, 15 and 30 mM] of NaP, as well as 2.5 and 10 μM of CH223191 and a combination of the above concentrations of NaP with10 μM of CH223191 over 48 h and control samples. Results [mean ± SD] were calculated as fold of control values. *p < 0.05, **p < 0.01 and ***p < 0.001, ANOVA.

Fig. 9A shows that concentrations of 15 and 30 mM NaA over 48 h significantly increased the expression of the *CYP1A1* gene (*P <* 0.05). Also, a concentration of 10 μM CH223191 significantly reduced the expression of the *CYP1A1* gene during this period (*P <* 0.05). However, a concentration of 2.5 μM CH223191 did not have a significant effect on reducing the expression of the *CYP1A1* gene. Furthermore, the combination of 7.5 mM, 15 mM and 30 mM NaA with a concentration of 10 μM CH223191 also reduced the expression of the *CYP1A1* gene. Fig. 9B shows that concentrations of 7.5 mM and 15 mM NaB over a 48- h period, significantly increased the expression of the *CYP1A1* gene (*P <* 0.05). Furthermore, a concentration of 10 μM CH223191 significantly reduced the expression of the *CYP1A1* gene during this period (*P <* 0.05). However, a concentration of 2.5 μM of CH223191 did not have a significant effect on reducing the expression of the *CYP1A1* gene. Combining a concentration of 30 mM NaB with a concentration of 10 μM CH223191 during this period resulted in a significant reduction in the *CYP1A1* gene expression. Similarly, the combination of 7.5 mM NaB with a concentration of 10 μM CH223191 also reduced the expression of the *CYP1A1* gene. Fig. 9C shows that concentrations of 7.5 mM, 15 mM and 30 mM NaP over a 48 −h period, significantly increased the expression of the *CYP1A1* gene (*P <* 0.05). Furthermore, a concentration of 10 μM CH223191 significantly reduced the expression of the *CYP1A1* gene during this period (*P <* 0.05). However, a concentration of 2.5 μM CH223191 did not have a significant effect on reducing the expression of the *CYP1A1* gene. Moreover, a combination of 7.5 mM, 15 mM and 30 mM NaP with a concentration of 10 μM CH223191 significantly reduced the expression of the *CYP1A1* gene.

### 3.5 Expression changes of the *CYP1A1* gene and MTT assay

**Fig. 10:**
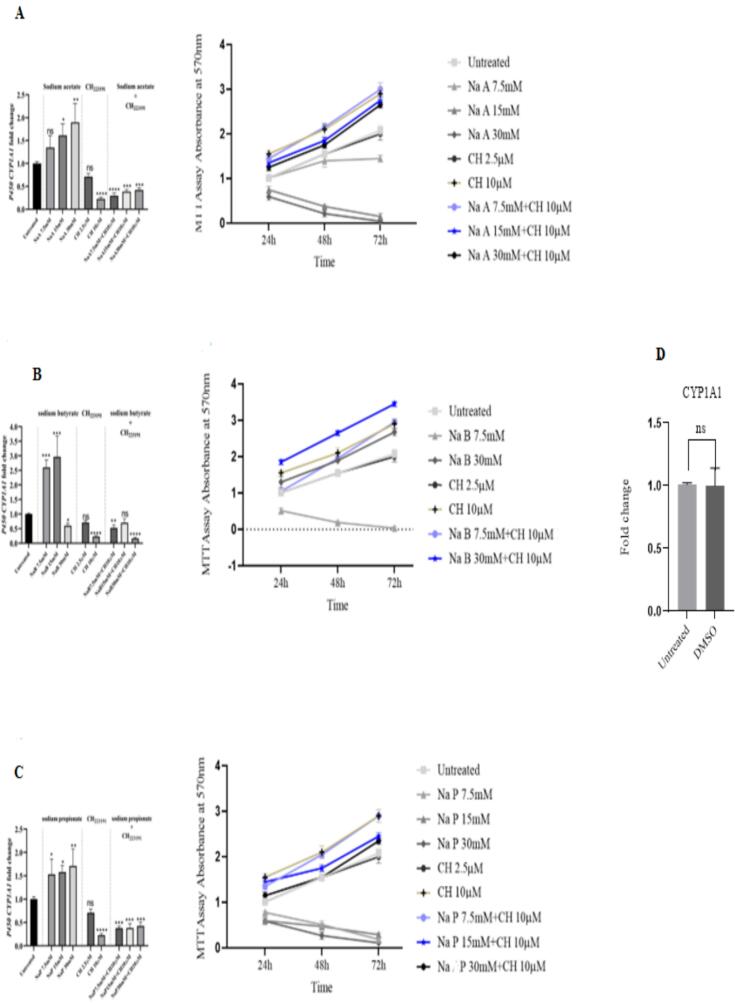
Expression changes of the *CYP1A1* gene and MTT assay. Cells were treated with various concentrations (7.5, 15, and 30 mM) of NaA (A), NaB (B), NaP (C), and DMSO (D) either in the absence or presence of CH223191.

A summary of the results is shown in Fig. 10. The compound CH223191 is an inhibitor of the AhR. Cells initially incubated with the SCFAs were then treated with CH223191 (10 μM) for 48 h. Incubation the cells with NaA (15 and 30 mM) for 48 h led to an increase in the expression of the *CYP1A1* gene and a decrease in cell proliferation (p < 0.05; Fig. 10A). NaB (7.5 and 15 mM) also showed a significant increase in *CYP1A1* gene expression (p < 0.05) and decreased cell proliferation (Fig. 10B). Treatment of cells with NaP (7.5, 15 and 30 mM) led to an increase in the expression of the *CYP1A1* gene and a decrease in cell proliferation (p < 0.05; Fig. 10C).

**Fig. 11:**
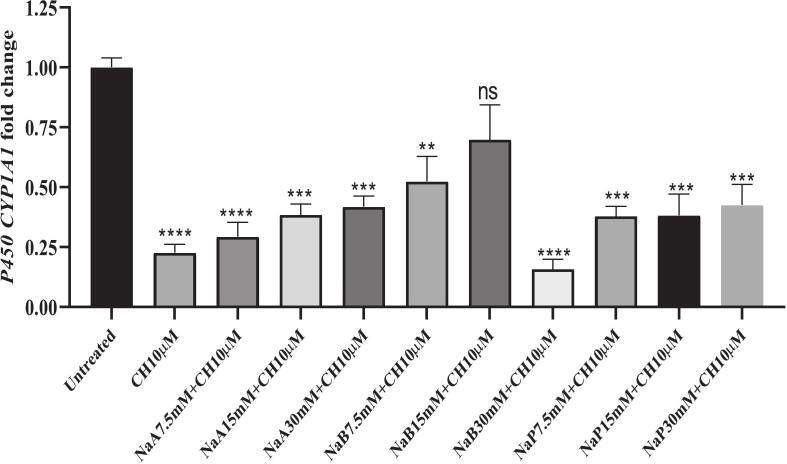
The effect of different concentrations of NaA, NaB and NaP combined at a concentration of 10 μM CH223191 on the expression of cytochrome P450*CYP1A1* gene has been investigated and compared. Asterisks indicate a substantial level compared to the untreated group. Results (mean ± SD) were calculated as fold of control values. *p < 0.05, **p < 0.01 and ***p < 0.001, ANOVA.

Treatment with CH223191(10 μM) significantly reduced the expression of the *CYP1A1* gene (*p <* 0.05), while a lower concentration of CH223191(2.5 μM) had no effect on either the expression of the *CYP1A1* gene or cell proliferation. Incubation with a combination of CH223191 (10 μM) and butyrate (15 mM) increased *CYP1A1* gene expression (p < 0.05), but could not fully reverse the inhibitory effect of CH223191 on the *CYP1A1* gene expression. In other words, this concentration of butyrate could only partially reduce the inhibitory effect of CH223191 on the *CYP1A1* gene expression. Fig. 11 shows that the lowest expression of the *CYP1A1* gene is related to the combination of 30 mM NaB and 10 μM CH223191 as well as 10 μM CH223191 alone. Also, concentrations of 7.5, 15 and 30-mM NaA and NaP in combination with 10 μM of CH223191 show a significant decrease in *CYP1A1* gene expression. However, a concentration of 15 mM NaB which significantly increases *CYP1A1* gene expression in combination with 10 μM of CH223191 did not significantly reduce expression compared to other samples.

### 3.6 Investigating the changes in the amount of CYP1A1 protein product by Western blot

**Fig. 12:**
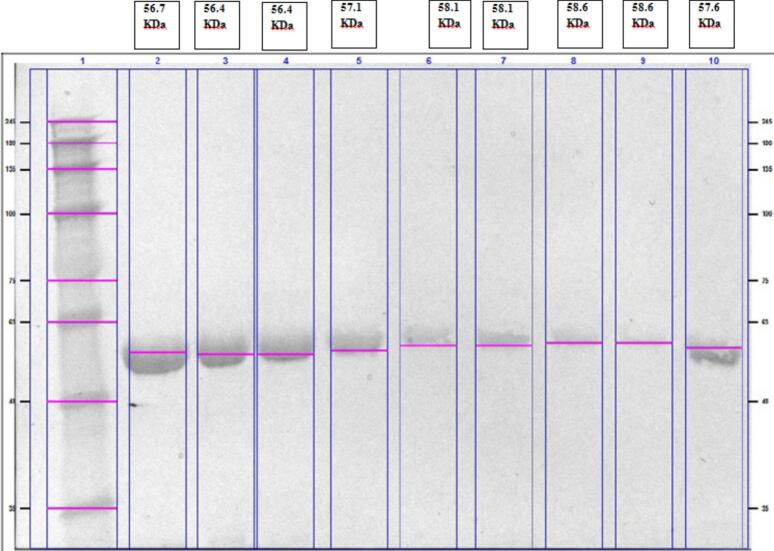
Investigating the increase in protein product of the *CYP1A1* gene, using Western blot in treated and non– treated cells. Lane 1: Ladder (35–48-63–75-100–135-180-245KDa), Lane2: NaB 24 h 15 Mm(56.7KDa), Lane3: NaP 24 h 30 mM(56.4KDa), Lane4: NaA 24 h 30 mM(56.4KDa), Lane5: NaA 24 h 7.5 mM(57.1KDa), Lane6: NaB 72 h 30 mM(58.1KDa), Lane7: NaA 48 h7.5 mM + CH223191 10 µM(58.1KDa), Lane8: CH223191 48h10µM(58.6KDa), Lane9: NaB 48 h 30 mM + CH223191 10 µM(58.6KDa), Lane10: control(57.6KDa).

**Fig. 13:**
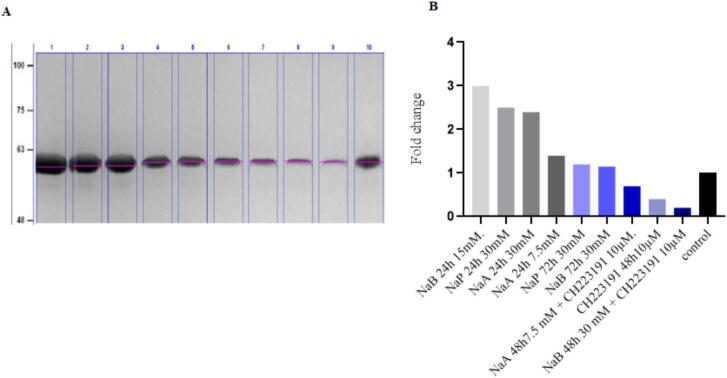
Investigating the increase in the protein product of the *CYP1A1* gene. A) Using Western blot in treated and untreated cells, 1) NaB 24 h 15 mM. 2) NaP 24 h 30 mM, 3) NaA 24 h 30 mM, 4) NaA 24 h 7.5 mM, 5) NaP 72 h 30 mM, 6) NaB 72 h 30 mM, 7) NaA 48 h7.5 mM + CH223191 10 µM, 8) CH223191 48h10µM, 9) NaB 48 h 30 mM + CH223191 10 µM, 10) control, B) Quantified plot of Western blot.

To confirm changes in gene expression using real-time PCR, we utilized the Western blot technique. This technique confirmed that the treated cells showed an increase in CYP1A1 gene expression, resulting in a higher production of the protein encoded by this gene (Fig. 12, Fig. 13).

**Fig. 14:**
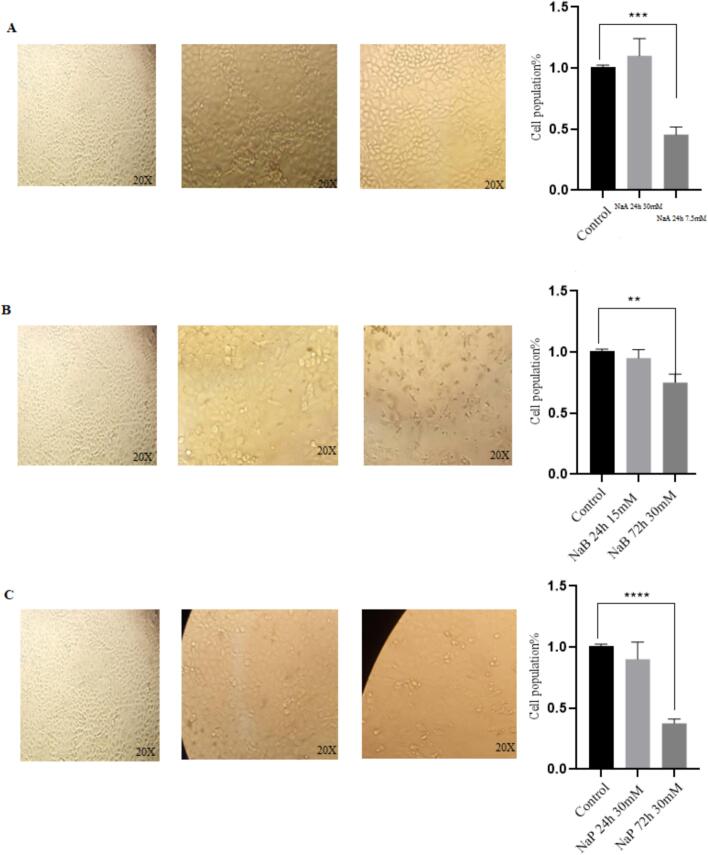
Morphologic evaluation of Caco-2 cells during various treatments. A, NaA in 24 h was used, with two concentrations and the period. The highest and lowest expression of the *CYP1A1* gene compared to other cell treatments with NaA at concentrations (7.5, 15 and 30 mM) and time intervals (24, 48 and 72 h) was seen. From left to right control, NaA 24 h 30 mM with the highest gene expression, NaA 24 h 7.5 mM with the lowest gene expression and a quantified plot of cell survival. In B, NaB at different time intervals in which in these two concentrations and periods, the highest and lowest expression of the *CYP1A1* gene compared to other cell treatments with NaB in concentrations (7.5, 15 and 30 mM) and time intervals (24, 48 and 72 h), have seen. From left to right control, NaB 24 h 15 mM with the highest gene expression, NaB 72 h 30 mM with the lowest gene expression and a quantified plot of cell survival. C, Sodium propionate at different time intervals was used, with two concentrations and periods. The highest and lowest expression of the *CYP1A1* gene compared to other cell treatments with sodium propionate at concentrations (7.5, 15 and 30 mM) and time intervals (24, 48 and 72 h), have seen. From left to right control, NaP 24 h 30 mM with the highest gene expression, NaP 72 h 30 mM with the lowest gene expression and a quantified plot of cell survival. Results (mean ± SD) were calculated as fold of control values. *p < 0.05, **p < 0.01 and ***p < 0.001, ANOVA.

Morphological investigation suggests that treating cells with specific short-chain fatty acids resulted in significant changes in the appearance of the treated cells compared to the control group. The control cells maintained their normal shape, while the treated cells showed deformation and cell death, which became more pronounced with increasing concentrations of these three substances. These three short-chain fatty acids can effectively influence the expression of genes involved in cell death and the *CYP1A1* gene. They also exhibit cytotoxicity on the Caco-2 strain at high IC50 concentrations. Comparing the morphology of treated cells to untreated cells (control group) reveals that increasing the concentration of treatments and incubation time leads to cancer cell death and increased expression of the *CYP1A1* gene. Treating cells with high concentrations of NaB and NaP is toxic and causes severe cell death, suggesting the induction of apoptosis at this concentration and time interval. Short-chain fatty acids, particularly butyrate, not only impact expression of the *CYP1A1* gene but can also trigger the expression of genes involved in apoptosis and cell death. Indeed, a direct relationship between the level of expression of the *CYP1A1* gene and the rate of cell death was observed (Fig. 14).

## 4 Discussion

The interaction between SCFAs and gut microbiota is complex and multifaceted. In a state of eubiosis, a diverse microbiota efficiently ferments dietary fibers into SCFAs. These SCFAs, in turn, support gut health by preserving the integrity of the intestinal barrier and regulating immune functions. However, when dysbiosis occurs characterized by reduced microbial diversity and an imbalance in microbial populations the production of SCFAs may be compromised.^7^, ^8^ This dysregulated fermentation process can lead to decreased levels of beneficial SCFAs and an increase in pro-inflammatory metabolites, contributing to the pathogenesis of various diseases. The results of this study demonstrate an increase in the expression of the *CYP1A1* gene in the Caco-2 cell line when treated with various concentrations of NaA, NaB, and NaP compared to the control group. Furthermore, there was a significant difference (p < 0.05) in gene expression between the control group and the treated cells. The protein level of gene expression was confirmed through Western blotting. Increasing the concentration of NaB, NaP, and NaA treatments below the IC50 led to an increase in the expression of the *CYP1A1* gene in the treated samples. Furthermore, at longer intervals and higher concentrations of NaB and NaP cell death was observed in the treated cells. Similar findings were seen for the *CYP1A1* gene expression and cell death, with NaA at concentrations higher than NaB, over the same period. Additionally, when combining NaB, NaP, and NaA at different concentrations with a concentration of 10 μM CH223191, a concentration of 15 mM NaB (which had the greatest effect on increasing the expression of the *CYP1A1* gene) in combination with 10 μM CH223191 did not show a notable decrease in expression of the *CYP1A1* gene compared to other treatments. This indicates that the anticancer effect of SCFAs depends on the concentration and duration of treatment. As the concentration and duration increase, the cytotoxic effect also increases, leading to cell death and decreased expression of the *CYP1A1* gene. SCFAs, especially butyrate, can also influence the expression of the *CYP1A1* gene, which is the target of this research, and can lead to the induction of genes involved in apoptosis and cell death. This suggests that high concentrations and long periods of time may not be suitable for examining the expression of the *CYP1A1* gene.

Forouzeh et al. (2021 and 2018) examined the expression of Bax and Bcl-2 genes in Caco-2 cells treated with varying concentrations of NaB. They found a significant increase in Bax expression and a decrease in Bcl-2 gene transcript expression. Their findings indicated that NaB, depending on the concentration and treatment duration, reduced the Bcl-2/Bax expression ratio and induce apoptosis in cancer cells.^42^, ^43^ Our results are consistent with their.

Marinelli (2018), aimed to identify alternative microbial species and metabolites that activate the AhR signaling pathway in human intestinal epithelial cells. He found that specific bacterial species were able to stimulate AhR signals by producing butyrate. This finding was successfully replicated in the Caco-2 intestinal epithelial cell line. Additionally, he observed that incubating intestinal epithelial cell lines with butyrate led to an increase in the expression of genes associated with AhR, AhRR, CYP1A1, and AhR protein.^44^

According to studies by Jin et al.(2017), SCFAs and other histone deacetylase inhibitors increase AhR-induced genes such as CYP1A1 by enhancing acetylation.^45^ Similar results were reported for propionate by Sheri Alex et al (2013).^46^ Propionate, like butyrate, significantly boots the transcriptional activity of PPARα^47^ and PPARβ.^48^ To demonstrate changes in gene expression in real-time PCR, Western blotting was used to confirm that in treated cells where expression of the *CYP1A1* gene was increased, the production of the protein encoded by this gene also increased. Our results align with studies by Wakx, A. (2018), where researchers confirmed through immunofluorescence and Western blotting that CYP1A1 protein levels follow the AhR activation pattern and are modulated by an AhR activator and inhibitor in VCT, BeWo, and NIH/3T3 cells.^49^

Also, Un-Ho Jin et al. (2017) examined the effect of butyrate, both alone and in combination with TCDD and three microbiota-derived AhR ligands (indole, tryptamine, and DHNA) on the expression of Ah-responsive genes, such as*TiPARP*, *AhRR*, *CYP1A1*, and *CYP1B1*.^50^ These genes are known to respond to TCDD, DHNA, and tryptophan metabolites. The researches observed that butyrate induced mRNA levels of CYP1B1 and CYP1A1 in both YAMC and Caco-2 cells, which was associated with increased histone acetylation (H3K9/14, H3K27, and H4K8) in both cell types. Similar effects on Ah-responsive genes were observed in Caco-2 and YAMC cells treated with propionate. Additionally, concentrations of acetate greater than 20 mM increased the expression of Ah-responsive genes and histone acetylation in both Caco-2 and YAMC cells.^51^

Central to the interaction between gut microbiota and the immune system are Toll-like receptors (TLRs). TLRs are pattern recognition receptors that play a pivotal role in the innate immune response by recognizing pathogen-associated molecular patterns (PAMPs) and damage-associated molecular patterns (DAMPs). The activation of TLRs triggers signaling cascades that result in the production of pro-inflammatory cytokines and chemokines, which are essential for mounting an effective immune response.^52^

Recent studies have highlighted the dual role of TLRs in cancer biology. On one hand, TLR activation can enhance anti-tumor immunity by promoting the maturation and activation of dendritic cells and stimulating T cell responses. On the other hand, chronic activation of TLRs due to dysbiosis or persistent inflammation can contribute to tumorigenesis by fostering an inflammatory microenvironment conducive to cancer development. This underscores the importance of a balanced gut microbiota in modulating TLR signaling pathways.^52^, ^53^ Moreover, SCFAs have been shown to influence TLR expression and function. For instance, butyrate has been reported to downregulate TLR4 expression in intestinal epithelial cells, thereby reducing inflammation and potentially lowering cancer risk. This intricate interplay between SCFAs, TLRs, and the immune system highlights a potential therapeutic avenue for cancer prevention and treatment through dietary modulation of gut microbiota.^54^

In conclusion, understanding the roles of TLRs in the context of gut microbiota and SCFAs provides valuable insights into their contributions to immune regulation and cancer pathogenesis. Future research should focus on elucidating the specific mechanisms by which TLRs mediate these interactions and exploring potential therapeutic strategies that harness the beneficial effects of SCFAs and a balanced gut microbiome in cancer prevention and treatment.

The aim of the present study was to investigate the interaction between gut microbiota metabolites and cancer cell biology, which is a very important area of ​​research. The use of multiple SCFAs at different concentrations and times, along with an AhR inhibitor, provides a comprehensive analysis of the research objectives. Additionally, the combination of MTT assay, qRT-PCR and Western blot analysis, along with multivariate validation of the findings, and appropriate statistical analysis are strengths of the present study. One limitation of this study was the use of only one cell line. In future studies, it would be beneficial to increase the number of cell lines studied. Additionally, incorporating mouse models and investigating the role of SCFAs in vivo could provide a more accurate representation of the offspring. Recent studies have highlighted the potential therapeutic benefits of SCFAs in restoring gut health and promoting eubiosis(7–10). For instance, dietary interventions aimed at increasing fiber intake can enhance SCFA production and support a healthy microbiota composition. Furthermore, supplementation with specific SCFAs has shown promise in ameliorating symptoms associated with dysbiosis-related conditions(8). Future research should focus on elucidating the precise mechanisms by which SCFAs influence gut microbiota dynamics and exploring targeted strategies to restore eubiosis in individuals with dysbiosis.

## 5 Conclusions

The results of this study demonstrate an increase in the expression of the *CYP1A1* gene in the Caco-2 cell line when treated with various concentrations of sodium acetate, sodium butyrate, and sodium propionate compared to the control group. Furthermore, there was a significant difference (p < 0.05) in gene expression between the control group and the treated cells. The protein level of gene expression was confirmed through Western blotting. Furthermore, examination of the treated cells revealed that the increased expression of the *CYP1A1* gene effectively inhibited proliferation and promoted cell death. These effects can be attributed to the inhibitory activity of SCFAs (acetate, butyrate, and propionate) on histone deacetylation. SCFAs are known to have anti-cancer and anti-proliferative effects on cancer cells. These findings provide an alternative perspective on the potential use of SCFAs, particularly acetate, butyrate, and propionate as epigenetic drugs for the prevention and treatment of colorectal cancer.

## CRediT authorship contribution statement

**Shiva Darabi:** Writing – original draft, Software, Resources, Methodology, Investigation, Funding acquisition, Formal analysis, Data curation. **Fatemeh Keshavarzi:** Writing – review & editing, Writing – original draft, Visualization, Validation, Supervision, Project administration, Methodology, Investigation, Data curation, Conceptualization. **Parviz Ashtari:** Validation, Supervision, Resources, Methodology, Investigation, Formal analysis, Data curation, Conceptualization. **Farahnaz Motamedi Sedeh:** Supervision, Software, Resources, Investigation, Formal analysis, Data curation, Conceptualization. **Behrouz Alirezapour:** Supervision, Software, Resources, Methodology, Investigation, Formal analysis, Data curation, Conceptualization.

## Declaration of Competing Interest

The authors declare that they have no known competing financial interests or personal relationships that could have appeared to influence the work reported in this paper.
